# Effects of cultivation of herbaceous perennials on soil microbial community structure and nitrogen cycling functions

**DOI:** 10.3389/fpls.2026.1961532

**Published:** 2026-10-02

**Authors:** Pushun Bian, Bo Han, Haifeng Zhu, Yu Zhou, Zexin Qi, Wei Gao, Yisen Wang, Nuo Chen

**Affiliations:** 1 College of Landscape Architecture, Changchun University, Changchun, China; 2 Agronomy College, Jilin Agricultural University, Changchun, China

**Keywords:** herbaceous perennials, metagenomics, microbial community, rhizosphere microecology, soil nitrogen cycling

## Abstract

This study aimed to elucidate the impacts of cultivating different herbaceous perennials on the rhizosphere microenvironment, nitrogen-cycling microbial communities, and functional genes in black soils of urban green spaces in Northeast China, thereby providing a scientific basis for rational urban plant configuration and interspecific differences in soil nutrient cycling. Focusing on five commonly utilized herbaceous perennials in Changchun (*Lycopus lucidus* [P-LL], *Salvia japonica* [P-SJ], *Hemerocallis fulva* [P-HF], *Hosta ensata* [P-HE], and *Pseudolysimachion spicatum* [P-PS]), this study integrated conventional physicochemical analyses, metagenomic sequencing, co-occurrence networks, and Random Forest modeling to analyze rhizosphere soil nutrient profiles, nitrogen-cycling microbial community structures, and key functional gene abundances after two years of continuous cultivation. Different floral treatments significantly altered rhizosphere nutrient status and microbial community composition. The P-LL treatment exhibited relatively higher contents of total nitrogen (TN), soil microbial biomass nitrogen (SMBN), available phosphorus, and available potassium, demonstrating a high-level soil multifunctionality index (SMI). The P-PS treatment showed higher available nitrogen and ammonium nitrogen but lower SMBN, accompanied by a higher relative abundance of Actinomycetota and the assembly of a microbial co-occurrence network with enhanced connectivity and robustness, indicating a potent organic nitrogen mineralization potential in its rhizosphere. The composition of nitrogen-cycling genes remained broadly stable across treatments, dominated by ammonia assimilation genes (*glnA* and *gltB*), with a relatively low abundance of denitrification genes (*nirK*). Multivariate and Random Forest analyses revealed that SMBN exerted the strongest positive regulatory effect on nitrogen-sensing genes (e.g., *ntrY* and *ntrX*), whereas TN, nitrate nitrogen (NO_3_^-^-N), and available potassium (AK) emerged as the paramount direct drivers determining the abundance variations of core ammonia assimilation genes. Different herbaceous perennials shaped species-specific rhizosphere nutrient and microbial functional profiles. P-LL and P-PS exhibited superior comprehensive nutrient traits and potential nitrogen mineralization characteristics, respectively, making them excellent candidate species for urban green space configuration and future mixed-planting trials in cold regions.

## 1 Introduction

Soil constitutes the core of terrestrial ecosystems, and its health status directly dictates plant growth and development. In the temperate continental monsoon climate zone of Northeast China, represented by urban ecosystems in Changchun City, Jilin Province, green space soils generally suffer from structural compaction and nutrient limitation. Consequently, the typical meadow black soils in these habitats often exhibit poor physicochemical properties and organic matter deficiency, which significantly impairs the underlying nutrient cycling efficiency and exacerbates nitrogen loss (Bender et al., 2023; Chukwujindu et al., 2026). During plant growth, nitrogen (N) is not only a vital nutrient for herbaceous perennials but also the primary limiting factor for the growth of most urban greening plants (Trammell et al., 2020). However, soil nitrogen typically cannot be directly and efficiently utilized by plants; it must undergo complex biogeochemical cycling processes to be converted into available nitrogen. The soil nitrogen cycling process is predominantly driven by soil microorganisms (Zheng et al., 2022). Therefore, considering the urban ecological characteristics of Northeast China, elucidating the plant-soil-microbe interaction mechanisms during nitrogen cycling is of paramount importance for guiding soil fertility maintenance and sustainable ecological construction in cold-region urban green spaces.

In Northeast China, represented by Changchun, traditional annual flowers are difficult to overwinter and incur high establishment costs. Herbaceous perennials are widely utilized in urban greening and landscape construction in Changchun due to their cold resistance, barren tolerance, and long life cycles (Celletti et al., 2025). During their growth, different herbaceous perennials alter the soil physicochemical microenvironment through root exudates and litter, exerting top-down effects that profoundly influence soil microbial community assembly and nutrient cycling (Koprivova and Kopriva, 2022). However, current greening practices in Northeast China predominantly focus on aboveground ornamental value and cold-resistance physiology, leaving a substantial knowledge gap regarding the interaction between belowground plant parts and soil microecology. The lack of a scientific understanding of how these plants regulate soil nitrogen cycling mechanisms can easily lead to localized nutrient imbalances. Thus, systematically investigating the effects of different herbaceous perennials in Northeast China on the microecological processes of soil nitrogen cycling holds significant practical and theoretical value for scientifically screening elite cultivars suitable for cold regions and optimizing urban soil nutrient management.

As the primary drivers of nutrient cycling, soil microorganisms directly affect plant growth and community succession (Trivedi et al., 2020). In complex soil microecosystems, nitrogen cycling is highly dependent on the orchestration of specific microbial functional groups; for instance, nitrification is dominated by genes such as *amoA*, while denitrification is regulated by genes including *nirK*, *nirS*, and *nosZ* (Li et al., 2025). Metagenomic sequencing, which targets the entire microbial genome, can effectively reveal community metabolic pathways and functional potentials within ecosystems. At present, research on the interaction mechanisms between plants and soil nitrogen cycling is primarily concentrated in agricultural and forest ecosystems, whereas studies specifically targeting herbaceous perennials remain scarce. In fact, one of the core driving forces of plant-soil interactions is that plant root exudates can disrupt the original soil carbon-nitrogen balance via the continuous input of active carbon (C), thereby triggering a strong rhizosphere priming effect (RPE). By activating specific microbial groups, this effect can significantly promote or inhibit the mineralization of soil organic matter, thereby determining the pulsed release (e.g., a surge) or immobilization of localized available nitrogen. However, existing research on flower microecology mostly remains confined to basic physicochemical indicators, failing to precisely locate these core functional groups involved in nitrogen cycling, and lacking a systematic network analysis of the interactions among environmental factors, plant phenotypes, and functional genes (Fan et al., 2023). Consequently, how different herbaceous perennials induce priming effects through the specific enrichment of their microecology, and subsequently regulate key nitrogen-cycling genes, remains entirely unknown.

To elucidate the aforementioned micro-driving mechanisms, this study focused on different herbaceous perennials under the habitat conditions of Changchun, Jilin, to systematically evaluate the impacts of plants on soil physicochemical properties and nutrient transformation. By integrating high-throughput metagenomic sequencing, co-occurrence networks, and machine learning models, we deeply profiled the response characteristics of soil nitrogen-cycling microbial community structures and their functional genes (nitrification and denitrification), and quantified the driving weights of core environmental factors on key metabolic pathways. Based on this, we hypothesize that different herbaceous perennials directionally recruit and screen specific key nitrogen-cycling microbial taxa (e.g., copiotrophic r-strategists or oligotrophic K-strategists) and reshape their network interaction patterns by constructing species-specific rhizosphere microenvironments. Furthermore, the consequent evolution of soil physicochemical properties serves as the core mechanism driving the directional changes in the abundance of these key functional genes. A systematic investigation into these microecological processes will facilitate a comprehensive understanding of environmental differences among various herbaceous perennials and their corresponding soil microecological remodeling traits. This provides robust theoretical guidance for evaluating the soil microecological optimization functions of floral species, scientifically selecting superior varieties suitable for cold regions, and managing urban green space soil nutrients.

## 2 Materials and methods

### 2.1 Description of the study area

The study was conducted in the experimental field at the West Campus of Changchun University in Changchun City, Jilin Province, China (125°18’E, 43°50’N), at an elevation of approximately 230 m. Situated in the hinterland of the Songnen Plain, the region is characterized by a mid-temperate continental monsoon climate. The mean annual temperature is 5.2 °C, and the mean annual precipitation reaches 570 mm. The annual effective accumulated temperature is approximately 2900 °C, and the annual effective solar radiation is 4750 MJ·m^-^². According to the classification standards of the Resource and Environment Science and Data Center, Chinese Academy of Sciences (CAS), the soil at the experimental site is classified as meadow black soil, with the texture being predominantly clay loam. This experimental site represents a typical urban green space soil habitat in Northeast China.

### 2.2 Sample collection and preparation

This experiment was conducted from May 2023 to September 2025. To eliminate the interference of pre-existing vegetation on soil physicochemical properties, all surface weeds were cleared in May 2023, and the plots were naturally air-dried for two weeks to serve as the planting substrate for each treatment. Five herbaceous perennials, characterized by natural variations in ecological habits and widely utilized in the urban landscaping of Changchun City, were selected as the tested plants: *Lycopus lucidus* (P-LL), *Salvia japonica* (P-SJ), *Hemerocallis fulva* (P-HF), *Hosta ensata* (P-HE), and *Pseudolysimachion spicatum* (P-PS).

A single-factor Randomized Complete Block Design (RCBD) was employed, establishing five monoculture treatments with three replicate blocks per treatment, yielding a total of 15 independent experimental plots. Each plot measured 2 m × 2 m, with a uniform planting density of 16 plants/m². In May 2023, robust primary seedlings of consistent age were transplanted into the experimental plots. Consistent conventional field management practices were implemented across all plots throughout the experiment. During the initial two weeks post-transplantation, uniform and moderate irrigation was applied to ensure seedling establishment; upon successful establishment, management was fully transitioned to a nature-dependent maintenance regimen. To prevent weeds from competing with the plants and interfering with soil properties, manual weeding was conducted regularly on a monthly basis during the experimental period. This maintained a strict monoculture status in each planting area, ensuring that the ultimate variations in soil physicochemical properties were exclusively driven by the physical penetration of the plant root systems and the ameliorative effects of their root exudates.

Sampling was conducted in September 2025, at which point all tested plants were fully developed, and the canopy coverage across all treatment plots had stably reached over 85%. To minimize sampling errors caused by soil spatial heterogeneity, an “S-shaped” five-point sampling method was adopted within each plot. Five representative plants with uniform growth were randomly selected, and their complete root systems within the 0–20 cm soil layer were carefully excavated. After gently shaking off the loosely attached bulk (non-rhizosphere) soil, the rhizosphere soil tightly adhering to the root surface (1–2 mm) was meticulously collected using a sterile brush, in strict compliance with aseptic operation protocols. Subsequently, the soil samples collected from the five points within the same plot were thoroughly pooled and homogenized to serve as a single independent biological replicate, resulting in three biological replicates per treatment (n = 3). After removing impurities such as animal and plant residues and gravel, the pooled samples were passed through a 2-mm sterile mesh sieve and equally divided into two portions. One portion was immediately snap-frozen in liquid nitrogen and transferred to a -80 °C ultra-low temperature freezer for subsequent metagenomic sequencing. The other portion was stored at 4 °C for the short-term determination of soil physicochemical properties and nutrient transformation indices.

### 2.3 Experimental methods

Soil pH was measured using the potentiometric method. Electrical conductivity (EC) was determined via the electrode method. Soil bulk density was measured using the cutting ring method, from which the soil water content and porosity were subsequently calculated.

Soil total nitrogen (TN) was determined using the modified Kjeldahl method (International Organization for Standardization, 1995). Soil samples were soaked in concentrated sulfuric acid, followed by digestion below 400 °C with the addition of a sodium thiosulfate reducing agent and a mixed catalyst. The digestion solution was then subjected to alkaline distillation; the distillate was absorbed by boric acid and titrated with a standard hydrochloric acid solution. A blank control was set simultaneously throughout the entire process to calculate the TN content.

Soil ammonium nitrogen (NH_4_^+^-N) and nitrate nitrogen (NO_3_^-^-N) contents were determined using the KCl extraction method (Keeney and Nelson, 1982). For NH_4_^+^-N, samples were shaken and extracted with a 2 mol/L KCl solution. After centrifugation, the supernatant was subjected to colorimetric analysis at a wavelength of 625 nm, and NH_4_^+^-N was quantified using a standard curve. For NO_3_^-^-N, fresh soil samples were extracted with a 1 mol/L KCl solution using the same procedure. Absorbance was measured at 220 nm and 275 nm, respectively, and the corrected absorbance was calculated using an empirical formula (A = A220 - 2.23 × A275) to eliminate the interference of dissolved organic matter, followed by NO_3_^-^-N quantification. Alkali-hydrolyzable nitrogen (AN) was measured using the alkaline hydrolysis diffusion method.

Soil microbial biomass nitrogen (SMBN) was measured using the chloroform fumigation-extraction-Kjeldahl method (Brookes et al., 1985). Ten milliliters of potassium chromium sulfate and 0.3 g of zinc powder were added to 30 mL of both fumigated and unfumigated extracts, followed by reduction at room temperature for 2 h. Subsequently, 0.6 mL of copper sulfate and 8 mL of concentrated sulfuric acid were added; the mixture was evaporated to dryness at 150 °C for 2 h and then subjected to high-temperature digestion for 3 h. Conventional alkaline distillation and titration were performed alongside a blank control. Finally, the total nitrogen content of the extracts was derived from the volume of standard acid consumed during titration. The SMBN content was accurately calculated by dividing the difference in total nitrogen between the fumigated and unfumigated soil samples by an extraction conversion factor (k_EN_), which is generally set to 0.54 (SMBN = EN/KEN).

Soil available phosphorus (AP) was determined using the Olsen method. Phosphorus-free activated carbon and a 0.5 mol/L NaHCO_3_ extraction solution (pH 8.5) were added to the sieved soil samples, followed by shaking extraction for 30 min and subsequent filtration. An appropriate amount of filtrate was mixed with a molybdenum-antimony-ascorbic acid color reagent. After standing for full color development, the absorbance was measured at 880 nm using a spectrophotometer, and quantitative calculation was completed in combination with a standard curve.

Soil available potassium (AK) was determined using the neutral ammonium acetate extraction method (Helmke et al., 1996). Sieved soil samples were mixed with a 1.0 mol/L ammonium acetate extraction solution (pH 7.0) at a soil-to-solution ratio of 1:10 (w/v) and extracted by shaking at a constant temperature of 25 °C for 30 min. After filtration, the potassium ion concentration in the extract was directly measured using a flame photometer and quantified via a standard curve.

Total soil DNA was extracted for metagenomic sequencing to construct a non-redundant gene catalog. The non-redundant protein sequences were aligned against the macro KEGG database (Kanehisa et al., 2023) and the specific NCyc database (Tu et al., 2019) using Diamond software (Buchfink et al., 2021) (alignment parameter: E-value ≤ 1e-5). Regarding the database integration strategy, the KEGG database was primarily employed to elucidate the overall dynamic characteristics of global metabolic pathways, whereas the NCycDB, a curated database specifically constructed for the nitrogen cycle, was prioritized for the precise localization and quantification of core nitrogen-cycling functional genes (e.g., *ntrY*, *glnA*, and *nirK*). To calculate gene abundance, high-quality reads from each sample were mapped back to the non-redundant gene catalog using Bowtie2 software. Gene abundance was normalized using the TPM (Transcripts Per Million) method. Given that TPM normalization mathematically eliminates biases caused by variations in gene length and sequencing depth across samples, and that the read coverage obtained via metagenomic sequencing directly reflects the allele frequencies within the DNA pool of environmental samples, this study directly utilized TPM abundance for subsequent inter-group comparisons, without applying the cross-species genomic copy number correction conventionally used in amplicon-based functional predictions.

Following the extraction of total soil DNA, a paired-end metagenomic sequencing library was constructed and sequenced on the Illumina NextSeq 2000 platform (Illumina, San Diego, CA, USA) under the PE150 mode by Shanghai Majorbio Bio-pharm Technology Co., Ltd. Quality control of the raw FASTQ files was performed using fastp v0.19.6 to remove low-quality reads—including those containing adapter sequences, >10% unknown nucleotides (N bases), or an average quality score below Q20—to obtain high-quality clean reads. Subsequently, co-assembly of the clean reads from each sample was conducted using MEGAHIT v1.2.9, and contigs with a length ≥ 300 bp were retained. Open reading frame (ORF) gene prediction was performed on the assembled contigs using Prodigal v2.6.3. The predicted protein-coding sequences were then subjected to homologous clustering using CD-HIT v4.8.1 (with a sequence identity threshold of 95% and an alignment coverage threshold of 90%) to remove redundancy and construct a non-redundant gene catalog. High-quality reads from each sample were mapped back to the non-redundant gene catalog using Bowtie2 v2.5.1, and gene abundances were normalized using the TPM method. For database alignment, Diamond v2.0.13 (E-value ≤ 1e-5, Identity ≥ 40%) was utilized to align the non-redundant protein sequences against the NR, KEGG (Kanehisa et al., 2023), and NCyc (Tu et al., 2019) databases for functional profiling and taxonomic annotation.

### 2.4 Data processing and statistical analysis

Metagenomic sequencing of the soil samples was performed by Shanghai Majorbio Bio-pharm Technology Co., Ltd. Bioinformatic analyses concerning the taxonomic composition and functional genes of nitrogen-cycling-related microorganisms were conducted using the Majorbio Cloud Platform. This platform was also utilized to perform redundancy analysis (RDA) and Spearman correlation analysis between the microbial communities and environmental factors. Additionally, the Linear discriminant analysis Effect Size (LEfSe) method was employed to identify statistically significant biomarker microbial taxa among the different treatment groups. This analysis initially utilized the non-parametric Kruskal-Wallis H test to detect species with significant differences in abundance among the groups (with the significance threshold set at *P* < 0.05). Subsequently, linear discriminant analysis (LDA) was applied to estimate the effect size of these differentially abundant taxa, with the logarithmic LDA score cutoff threshold set at > 2.0.

Statistical analyses of the conventional soil physicochemical properties and nutrient transformation data were performed using SPSS 25.0 software. One-way analysis of variance (ANOVA) combined with Duncan’s multiple range test (*P* < 0.05) was used to analyze the significant differences in soil microenvironment physicochemical indicators, nutrient contents, and microbial α-diversity following the planting of different herbaceous perennials. For the relative abundance of specific functional genes that did not meet the assumptions of normal distribution and homogeneity of variance, the non-parametric Kruskal-Wallis H test was adopted for multigroup significance comparisons. In the β-diversity analysis, principal coordinate analysis (PCoA) based on distance matrices was conducted, and the non-parametric Analysis of Similarities (ANOSIM) test was employed to evaluate the significant differences in community structures across different treatments. For the RDA, a Monte Carlo permutation test with 999 permutations was used to assess the statistical significance of the explanatory power of environmental factors on the distribution of microorganisms and functional genes.

Furthermore, to comprehensively evaluate the specific impacts of different herbaceous perennials on the overall soil nutrient cycling functions, the Soil Multifunctionality Index (SMI) was calculated based on the Z-score standardization method to synthetically assess the overall nutrient cycling capabilities of the different treatments. Specifically, seven core physicochemical and biological indicators (TN, NH_4_^+^-N, NO_3_^-^-N, SMBN, AN, AP, and AK) for each sample were individually transformed using Z-scores to eliminate dimensionality. The SMI was then derived by calculating the mean of the standardized values for each sample. Data processing and data visualization were completed using Origin 9.0 software. Data in the conventional graphs and tables are presented as the “mean ± standard deviation (Mean ± SD)”. To further quantify the relative impact of soil physicochemical factors on the abundance of key nitrogen-cycling functional genes, a Random Forest model was employed. The Increase in Mean Squared Error (%IncMSE) was utilized as the variable importance evaluation metric, where a higher %IncMSE value indicates a more significant direct or indirect driving effect of a given factor on the abundance distribution of specific functional genes.

A co-occurrence network was constructed based on the relative species abundance at the genus level to investigate the effects of different herbaceous perennial treatments on the interactions and stability of the microbial community. Spearman correlation analysis was applied to calculate interspecific correlation coefficients, retaining only significantly correlated edges with |r| > 0.6 and P < 0.05. Network topological properties, primarily including average degree, modularity, and robustness, were calculated using the *igraph* package.

## 3 Results and analysis

### 3.1 Effects of different herbaceous perennials on soil physicochemical properties and nutrient transformations

Prior to the initiation of the experiment in May 2023, the homogenized basal soil across the experimental plots was uniformly measured to establish the initial baseline data: pH, 7.5; soil moisture content (SMC), 8.1%; total nitrogen (TN), 0.8 g/kg; alkali-hydrolyzable nitrogen (AN), 72.2 mg/kg; available phosphorus (AP), 84.8 mg/kg; and available potassium (AK), 113.1 mg/kg. These baseline data confirmed that all treatment plots possessed a highly consistent physicochemical background prior to planting, thereby providing a reliable time-zero reference for evaluating the species-specific effects induced by the establishment of herbaceous perennials over the subsequent two years.As presented in **Table 1**, the different herbaceous perennial treatments significantly affected the soil physicochemical properties and nutrient contents (*P* < 0.05). The soils across all treatments exhibited slight alkalinity, with pH values ranging from 7.33 to 7.53. The average SMC was 9.88%; notably, the P-HF treatment recorded the highest value (13.91%), whereas the P-PS treatment registered the lowest (5.07%). The average contents of soil TN, NH_4_^+^-N, and NO_3_^-^-N were 0.81 g/kg, 9.22 mg/kg, and 3.39 mg/kg, respectively. The TN content (1.22 g/kg) and SMBN levels in the P-LL treatment were significantly higher than those in the other treatments. The P-PS treatment yielded the highest NH_4_^+^-N and AN contents, reaching 10.22 mg/kg and 120.96 mg/kg, respectively; meanwhile, the NO_3_^-^-N content peaked under the P-HE treatment (6.26 mg/kg). The AK content achieved its maximum value in the P-LL treatment (194.43 mg/kg). Additionally, the AP content in the P-LL treatment (94.48 mg/kg) was at the same significance level as that in the P-HE treatment (93.57 mg/kg), with both being significantly higher than the other treatments.The Soil Multifunctionality Index (SMI), calculated based on individual nutrient indicators, demonstrated that the different herbaceous perennials significantly altered the overall functional potential of the rhizosphere microenvironment. The P-LL treatment exhibited the highest SMI value (0.58), significantly outperforming the other treatments. The SMI value of the P-PS treatment ranked second (0.10); this was primarily driven by its prominent performance in available nitrogen (AN and NH_4_^+^-N) activation, though its overall multifunctionality was constrained by an exceptionally low SMBN level. The SMI values for the P-HE, P-HF, and P-SJ treatments were relatively low, recorded at -0.03, -0.27, and -0.38, respectively. Overall, the different herbaceous perennials influenced soil nutrients through distinct pathways. The P-LL treatment maintained a relatively high comprehensive soil nutrient profile, whereas the P-PS treatment displayed a more abundant available nitrogen pool.

**Table 1:** Effects of different herbaceous perennials on soil physicochemical properties and nutrient contents.

No.	pH	SMC(%)	TN (g·kg^-1^)	NH_4_^+^-N(mg·kg^-1^)	NO_3_^-^-N(mg·kg^-1^)	SMBN(mg·kg^-1^)	AN (mg·kg^-1^)	AP (mg·kg^-1^)	AK (mg·kg^-1^)
P-LL	7.33 ± 0.05d	10.75 ± 1.06ab	1.22 ± 0.01a	8.66 ± 1.05bc	3.70 ± 1.93bc	62.65 ± 0.03a	85.90 ± 1.39b	94.48 ± 0.99a	194.43 ± 0.06a
P-SJ	7.42 ± 0.01bc	12.45 ± 4.41a	0.71 ± 0.00e	9.62 ± 0.26ab	0.21 ± 0.04d	51.12 ± 0.19e	50.89 ± 1.58d	47.71 ± 0.51d	161.50 ± 0.10c
P-HF	7.34 ± 0.02cd	13.91 ± 2.20a	0.57 ± 0.01f	8.98 ± 0.04bc	1.72 ± 0.12cd	56.00 ± 0.12d	50.03 ± 1.28d	73.77 ± 1.43c	152.37 ± 0.21d
P-HE	7.53 ± 0.05a	7.24 ± 0.38bc	0.81 ± 0.01c	8.62 ± 0.51bc	6.26 ± 2.03a	61.65 ± 0.09b	73.63 ± 1.18c	93.57 ± 0.69a	129.83 ± 0.06e
P-PS	7.51 ± 0.06a	5.07 ± 1.16c	0.75 ± 0.01d	10.22 ± 0.48a	5.08 ± 1.83ab	32.06 ± 0.12f	120.96 ± 1.32a	74.17 ± 2.30c	175.23 ± 0.35b
p-value	<0.05	<0.05	<0.05	<0.05	<0.05	<0.05	<0.05	<0.05	<0.05

SMC, soil moisture content; TN, total nitrogen; NH₄⁺-N, ammonium nitrogen; NO₃⁻-N, nitrate nitrogen; SMBN, soil microbial biomass nitrogen; AN, available nitrogen; AP, available phosphorus; AK, available potassium. Different lowercase letters indicate significant differences among treatments at the 0.05 level. The same below.

### 3.2 Differences in soil nitrogen-cycling microbial community structure and functional genes among different herbaceous perennials

#### 3.2.1 Alpha diversity of nitrogen-cycling microorganisms and functional genes

As shown in **Table 2**, the sequence coverage of soil nitrogen-cycling microbial communities under different herbaceous perennial treatments was uniformly 1, with no significant differences, indicating that the sequencing data adequately reflected the composition and structural distribution characteristics of the microbial communities. Regarding the richness indices, the P-SJ treatment exhibited the highest Sobs, ACE, and Chao1 indices (177.00), while the Shannon, Simpson, and Pielou_e indices showed highly significant differences among the treatments (*P* < 0.001). The P-HE treatment displayed the highest Shannon index (4.144), whereas the P-PS treatment presented the lowest diversity and evenness. This demonstrates that different herbaceous perennials significantly influenced the richness and community structure of soil nitrogen-cycling microorganisms.

**Table 2:** Alpha diversity indices of soil nitrogen-cycling microorganisms under different herbaceous perennial treatments.

No.	Sobs, ace, chao1	Shannon	Simpson	Pielou_e	coverage
P-LL	170.00 ± 8.00ab	4.054 ± 0.028bc	0.034 ± 0.001bc	0.789 ± 0.012b	1
P-SJ	177.00 ± 6.25a	4.107 ± 0.014ab	0.032 ± 0.001c	0.793 ± 0.003b	1
P-HF	163.00 ± 7.21b	4.041 ± 0.047c	0.037 ± 0.003b	0.793 ± 0.003b	1
P-HE	165.67 ± 3.22b	4.144 ± 0.034a	0.031 ± 0.000c	0.811 ± 0.007a	1
P-PS	163.00 ± 2.65b	3.901 ± 0.037d	0.044 ± 0.004a	0.766 ± 0.005c	1
p-value	<0.05	<0.001	<0.001	<0.001	—

#### 3.2.2 Beta diversity of nitrogen-cycling microorganisms and functional genes

**Figure 1** illustrates the principal coordinate analysis (PCoA) of the soil nitrogen-cycling microbial communities under various treatments, demonstrating the differences in beta diversity among the samples. The first (PC1) and second (PC2) principal components accounted for 72.68% and 10.66% of the total variance, respectively. With a cumulative contribution of 83.34%, these axes effectively capture the structural disparities in the microbial communities among the samples. Based on the spatial distribution of the sample coordinates in the PCoA plot and the accompanying statistical validation (R = 0.928, P = 0.001), the replicates within each treatment group clustered closely together, whereas the clusters representing different treatments were distinctly separated. This indicates significant differences in the composition of the soil nitrogen-cycling microbial communities across the different treatments. The coordinates for the P-SJ treatment were positioned along the positive axis of PC1, exhibiting the greatest distance from the other treatment groups, which suggests that it harbored the most distinct community structure. Conversely, the coordinates for both the P-LL and P-PS treatments were situated along the negative axis of PC1 and clustered in close proximity to each other, reflecting a high degree of similarity in their microbial community compositions. The high degree of intra-group clustering observed in the P-HF and P-SJ treatments indicates substantial community homogeneity and a relatively stable micro-ecological environment. In contrast, the greater dispersion of sample coordinates within the P-HE, P-PS, and P-LL groups highlights more pronounced intra-group variation. Furthermore, the sample coordinates for the P-HE and P-HF treatments aggregated in the upper and lower quadrants of the plot, respectively, demonstrating a clear separation along the PC2 axis. In conclusion, the species of herbaceous perennials significantly drove the differentiation of the soil nitrogen-cycling microbial communities and profoundly influenced the spatial stability of these communities within the respective groups.

**Figure 1:**
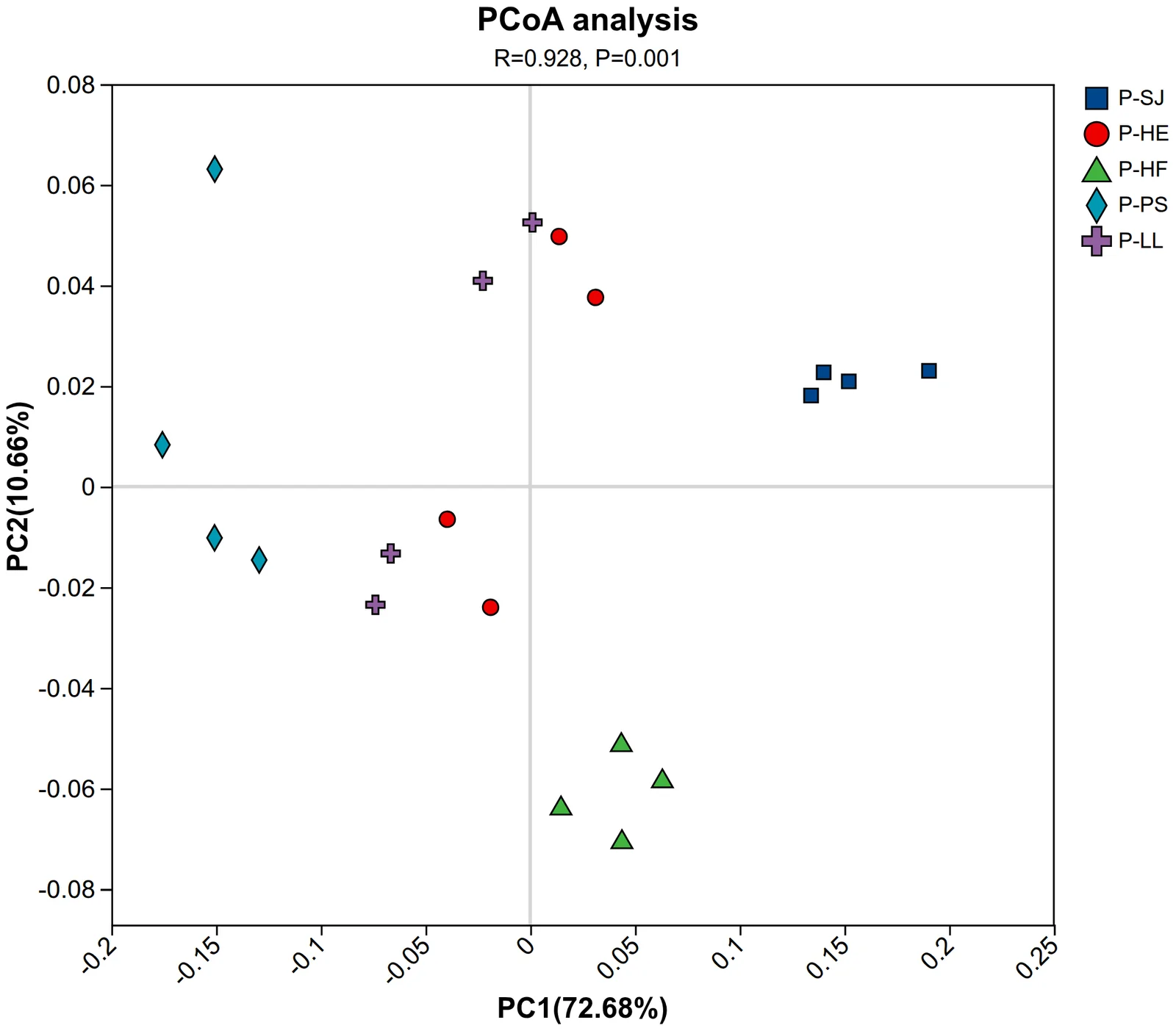
Principal Coordinate Analysis (PCoA) of soil nitrogen-cycling microbial communities under different herbaceous perennials.

#### 3.2.3 Species composition of nitrogen-cycling functional microorganisms

**Figure 2** illustrates the taxonomic composition of the soil nitrogen-cycling microbial communities at the phylum level across the different treatments. Overall, the composition of the dominant microbial phyla remained consistent across the five herbaceous perennial treatments, although their relative abundances varied. Notably, Pseudomonadota exhibited absolute dominance in all treatments, accounting for the highest relative abundance (ranging from 35.7% to 61.3%). This was followed by Actinomycetota and Acidobacteriota, with average relative abundances of approximately 19.5% and 14.2%, respectively. The proportional abundances of these communities varied markedly among the treatments. The relative abundance of Pseudomonadota peaked in the P-SJ treatment at 61.3%. In contrast, the P-HE, P-HF, P-PS, and P-LL treatments displayed higher relative abundances of Actinomycetota and Acidobacteriota compared to the P-SJ treatment, with this trend being particularly pronounced in the P-PS and P-LL treatments. Additionally, taxa such as Nitrososphaerota and Gemmatimonadota exhibited relatively low abundances.

**Figure 2:**
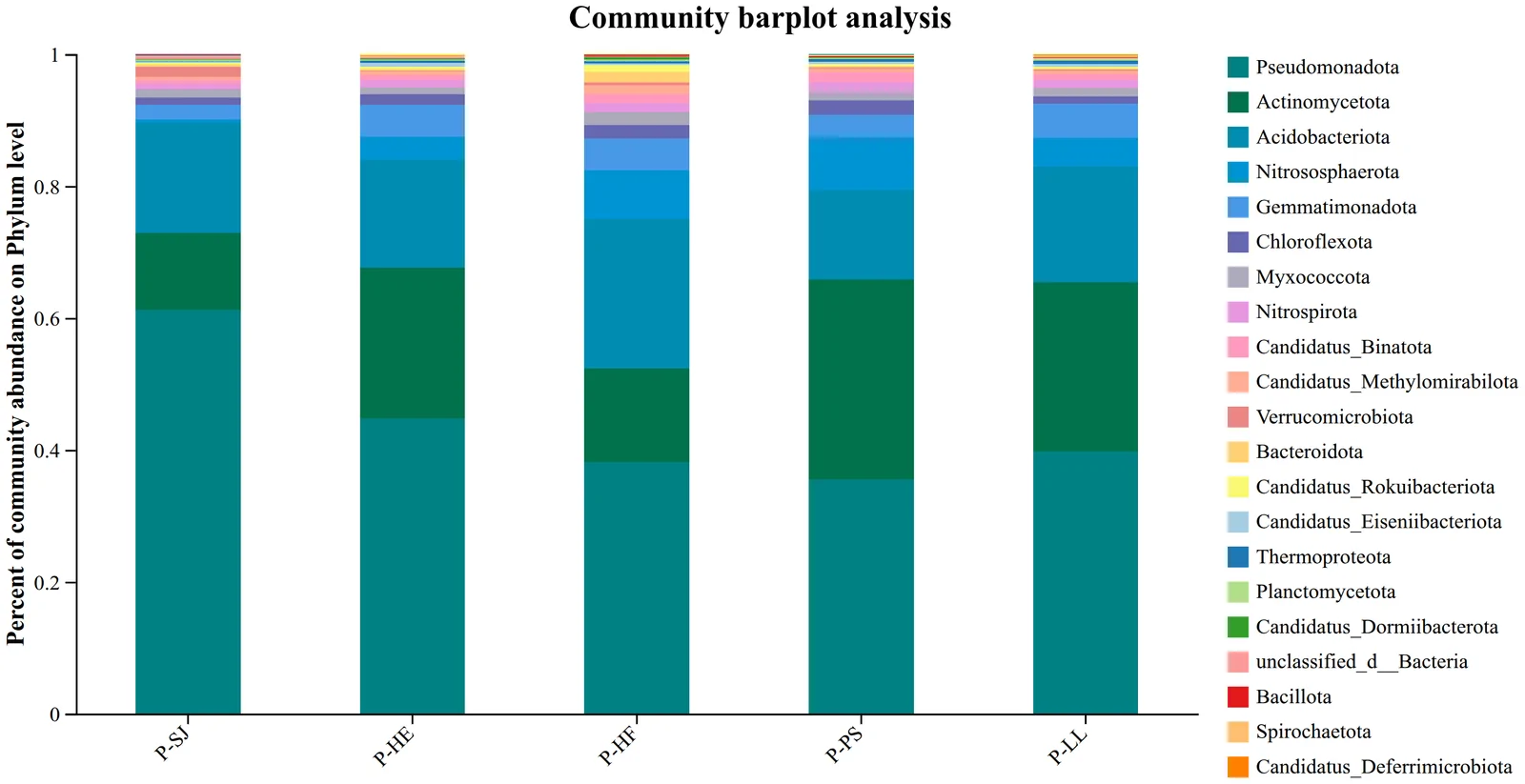
Relative abundance of soil nitrogen-cycling microbial communities at the phylum level under different herbaceous perennials.

The LEfSe analysis (**Figure 3**), conducted with an expected value threshold of *P* < 0.05 and a logarithmic LDA score > 2.0, revealed numerous statistically significant indicator clades of nitrogen-cycling microorganisms, spanning from the phylum to the genus level, across the distinct rhizosphere environments of the different herbaceous perennials.

**Figure 3:**
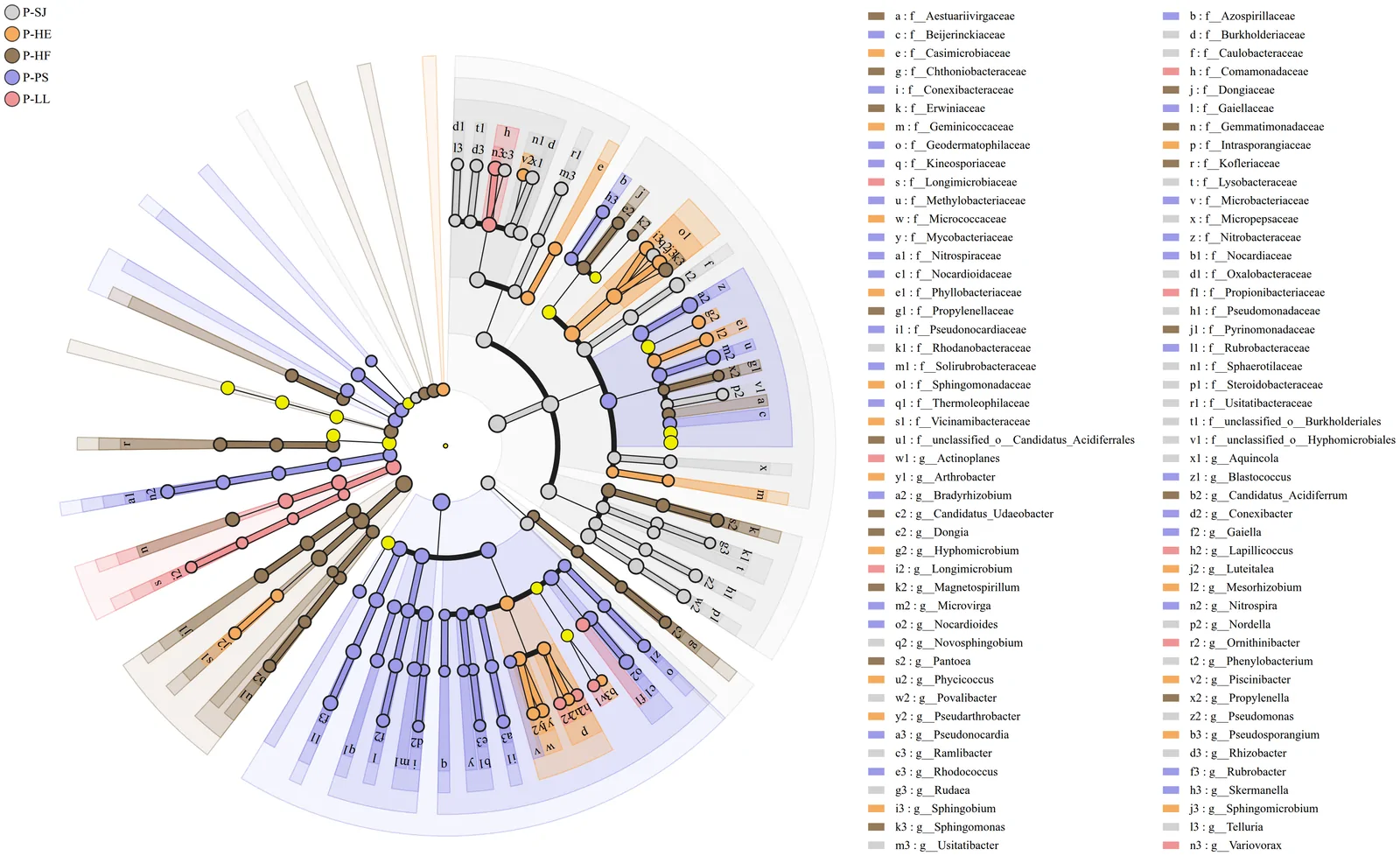
Linear discriminant analysis effect size (LEfSe) of soil nitrogen-cycling microbial communities under different herbaceous perennials.

Specifically, the P-PS treatment exhibited higher enrichment levels in several major clades within the phylum Actinomycetota. As typical K-strategists, members of Actinomycetota possess robust metabolic capacities for degrading complex organic carbon, such as lignin and cellulose. Furthermore, their decomposition of complex carbon sources is frequently tightly coupled with the mineralization and release of soil organic nitrogen. This suggests that the P-PS roots exuded structurally complex carbon sources into the soil, which not only conferred a critical competitive advantage to Actinomycetota but also accelerated the turnover of organic nitrogen, thereby establishing a microbiological foundation for the subsequent rhizosphere priming effect.In the P-HF treatment, the enriched taxa were predominantly concentrated within the phyla Acidobacteriota and Gemmatimonadota. Conversely, the P-SJ and P-HE treatments demonstrated elevated characteristics in distinct clades within the phylum Pseudomonadota. Pseudomonadota primarily comprise fast-growing, r-strategy copiotrophic bacteria that rapidly respond to easily accessible labile carbon, indicating a more active metabolism of root exudates within the P-SJ and P-HE treatments. Collectively, these findings demonstrate that the species-specific rhizosphere microenvironments of the diverse herbaceous perennials profoundly shape the taxonomic community structure of key nitrogen-cycling microorganisms.

#### 3.2.4 Co-occurrence networks and community stability of nitrogen-cycling microorganisms

Phylum-level co-occurrence network analysis revealed distinct interaction patterns within the microbial communities across different herbaceous perennial treatments (**Figure 4**). Comparisons of topological properties demonstrated a divergence in community stability and complexity among the treatments. Notably, the microbial network under the P-PS treatment exhibited a relatively higher average degree and robustness, featuring a larger proportion of positive correlations, which indicates stronger connectivity among the nodes. Furthermore, the network under the P-LL treatment also displayed a higher modularity index. In contrast, the network structures of certain other treatments were relatively simplified, exhibiting lower connectivity. These results indicate that the cultivation of different herbaceous perennials significantly altered the co-occurrence patterns among core microbial taxa and the overall structural stability of the communities.

**Figure 4:**
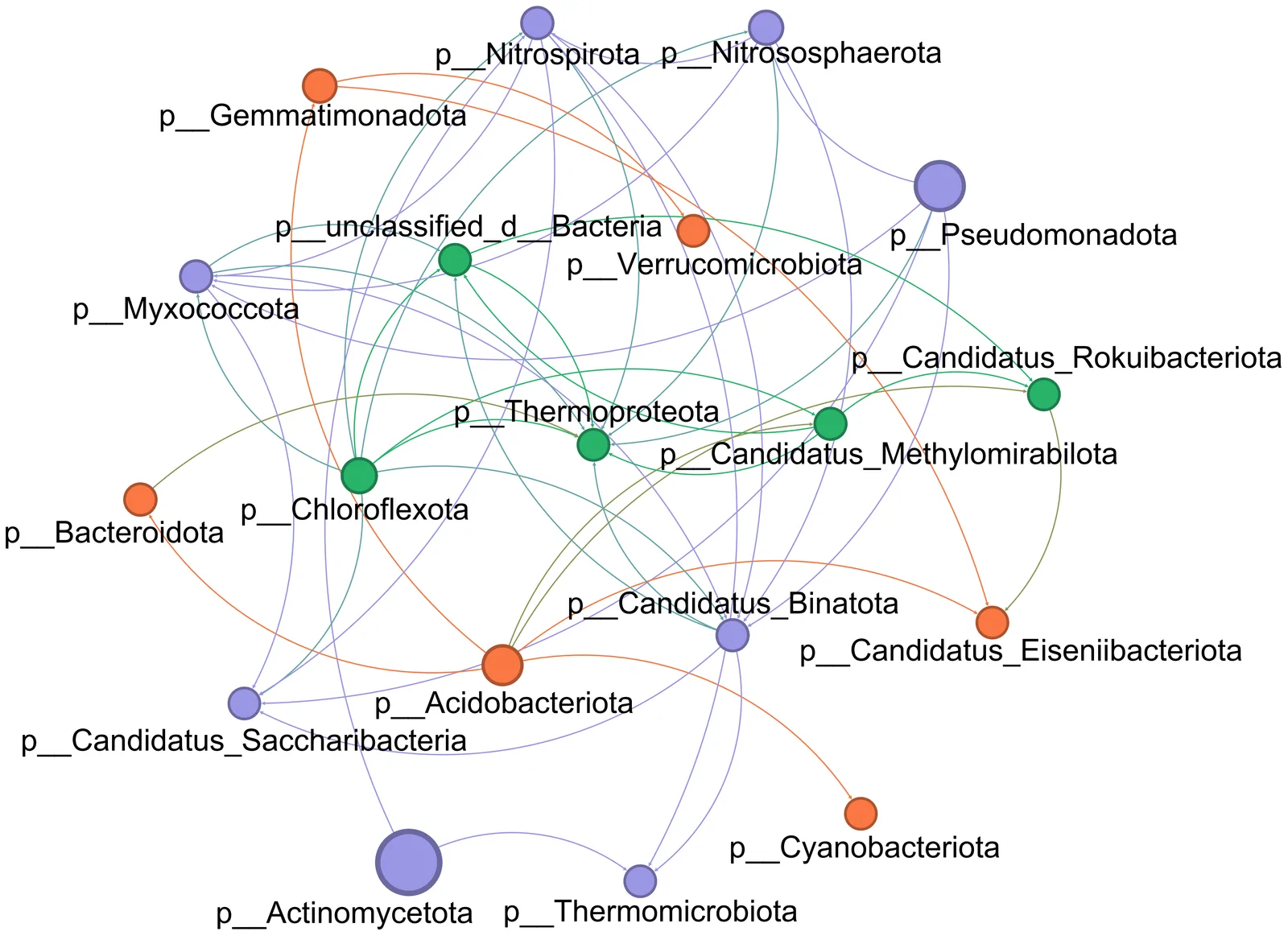
Co-occurrence networks and topological properties of soil microorganisms under different herbaceous perennials.Note: Different colors represent different modules, and node sizes indicate the degree of connectivity.

#### 3.2.5 Abundance of nitrogen-cycling functional genes

**Figure 5** illustrates the differences in the relative abundance of the core soil nitrogen regulation gene *ntrY* across the different herbaceous perennial treatments. The results revealed significant overall variations in the abundance of this gene among the floral treatments, with the P-HF and P-PS treatments exhibiting the highest and lowest relative abundances of *ntrY*, respectively. Notably, the boxplots for the P-PS and P-SJ treatments displayed broader distribution ranges, indicating substantial variation in gene abundance among the replicate samples within these treatments and revealing strong intra-group heterogeneity. Conversely, the box distributions for the P-HE and P-HF treatments were relatively compact, suggesting lower intra-group data variance.Furthermore, multiple comparisons demonstrated significant differences in relative gene abundance across the groups. Specifically, the abundance in the P-PS treatment was highly significantly lower than those in the P-SJ, P-HE, and P-HF treatments (*P* < 0.001), and significantly lower than that in the P-LL treatment (*P* < 0.05). Meanwhile, the P-HF treatment was highly significantly greater than the P-LL treatment (*P* < 0.001) and significantly higher than the P-HE treatment (*P* < 0.05). These findings indicate that the species-specific rhizosphere microenvironments of the diverse herbaceous perennials distinctively shape the abundance of key soil nitrogen regulation genes. The relative abundance of the *ntrY* gene peaked significantly under the P-HF treatment, whereas the P-PS samples exhibited an overall lower abundance accompanied by pronounced internal fluctuations.

**Figure 5:**
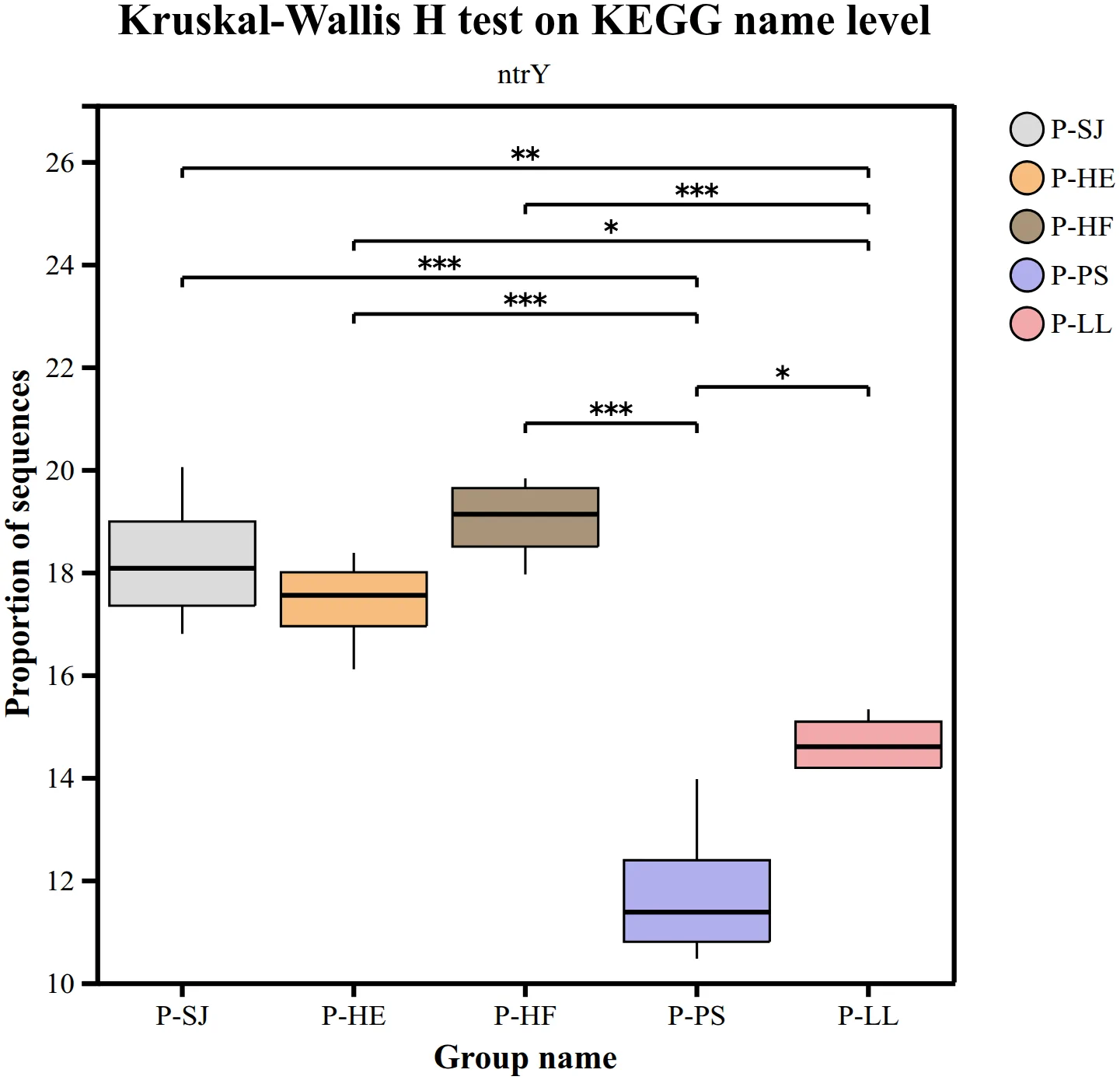
Abundance of the nitrogen-cycling functional gene (*ntrY*, nitrogen regulation sensor histidine kinase) under different herbaceous perennials. * indicates P < 0.05, ** indicates P < 0.01, and *** indicates P < 0.001.

### 3.3 Effects of different herbaceous perennials on the abundance of key functional genes involved in nitrogen metabolism

As illustrated in **Figure 6**, the soil bacterial communities under different herbaceous perennials were predominantly involved in several key nitrogen metabolism pathways, including ammonia assimilation and urea metabolism, denitrification, assimilatory nitrate reduction, as well as dissimilatory nitrate reduction and nitrification.

**Figure 6:**
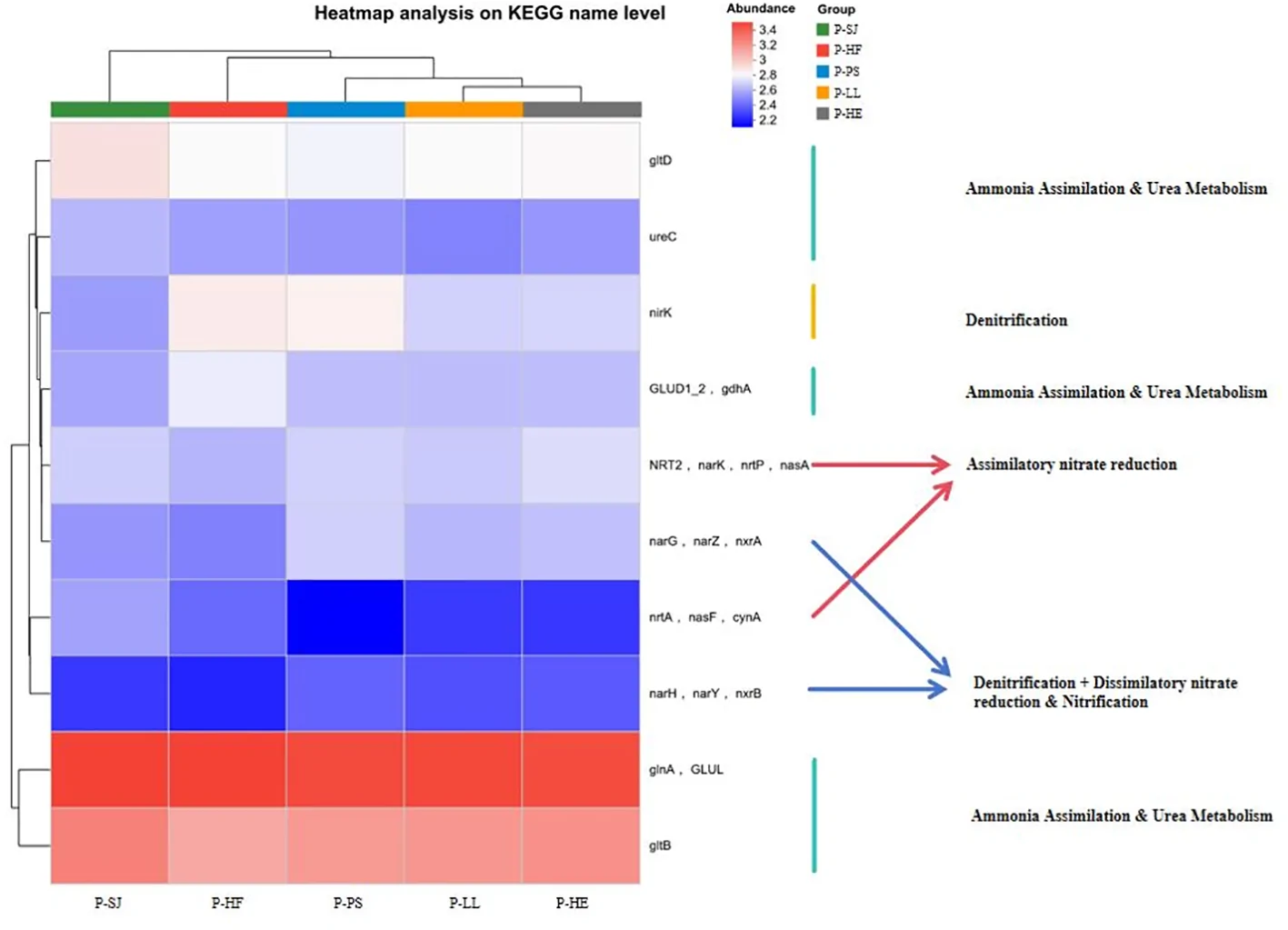
Heatmap analysis of the abundance of nitrogen metabolism-related genes in soil bacterial communities under different herbaceous perennials.

Overall, the dominant nitrogen metabolism processes exhibited high consistency across the different plant treatments, although the relative abundances of distinct functional genes differed significantly. Soil microorganisms under all treatments were dominated by ammonia assimilation and urea metabolism pathways. Specifically, genes encoding glutamine synthetase (*glnA* and *GLUL*) and glutamate synthase (*gltB*) exhibited the highest relative abundances, consistently maintaining remarkably elevated levels across all herbaceous perennial treatments. Nevertheless, treatment-specific divergence was also observed. In the soil of the P-SJ treatment, the relative abundance of the *gltD* gene, which participates in the ammonia assimilation pathway, was markedly higher than that in other treatments. In the P-HF treatment, the relative abundances of key denitrification genes (*nirK*), assimilatory nitrate transporter genes (*NRT2*, *narK*, *nrtP*, and *nasA*), and glutamate dehydrogenase genes involved in ammonia assimilation (*GLUD1_2* and *gdhA*) displayed varied degrees of elevation compared to the other groups. In contrast, genes encoding nitrate/nitrite transporters (*nrtA*, *nasF*, and *cynA*) and those involved in denitrification and nitrification (*narH*, *narY*, and *nxrB*) generally maintained lower relative abundances across all treatments, reaching their lowest levels in the P-PS, P-LL, and P-HE treatments.

### 3.4 Correlation analysis between soil chemical properties and nitrogen-cycling functional genes

As shown in **Figure 7**, significant differences were observed in the relative abundances of soil nitrogen-cycling-related functional genes across the different herbaceous perennial treatments. The Kruskal-Wallis H test results revealed that *gltB* and *gltD* exhibited highly significant enrichment in the P-SJ treatment (*P* < 0.01), whereas *GLUD1_2 gdhA* and *nirK* reached their highest relative abundances in the P-HF treatment. This indicates that plant species exert a pronounced filtering and reshaping effect on the genetic composition and abundance of soil nitrogen-cycling microorganisms.

**Figure 7:**
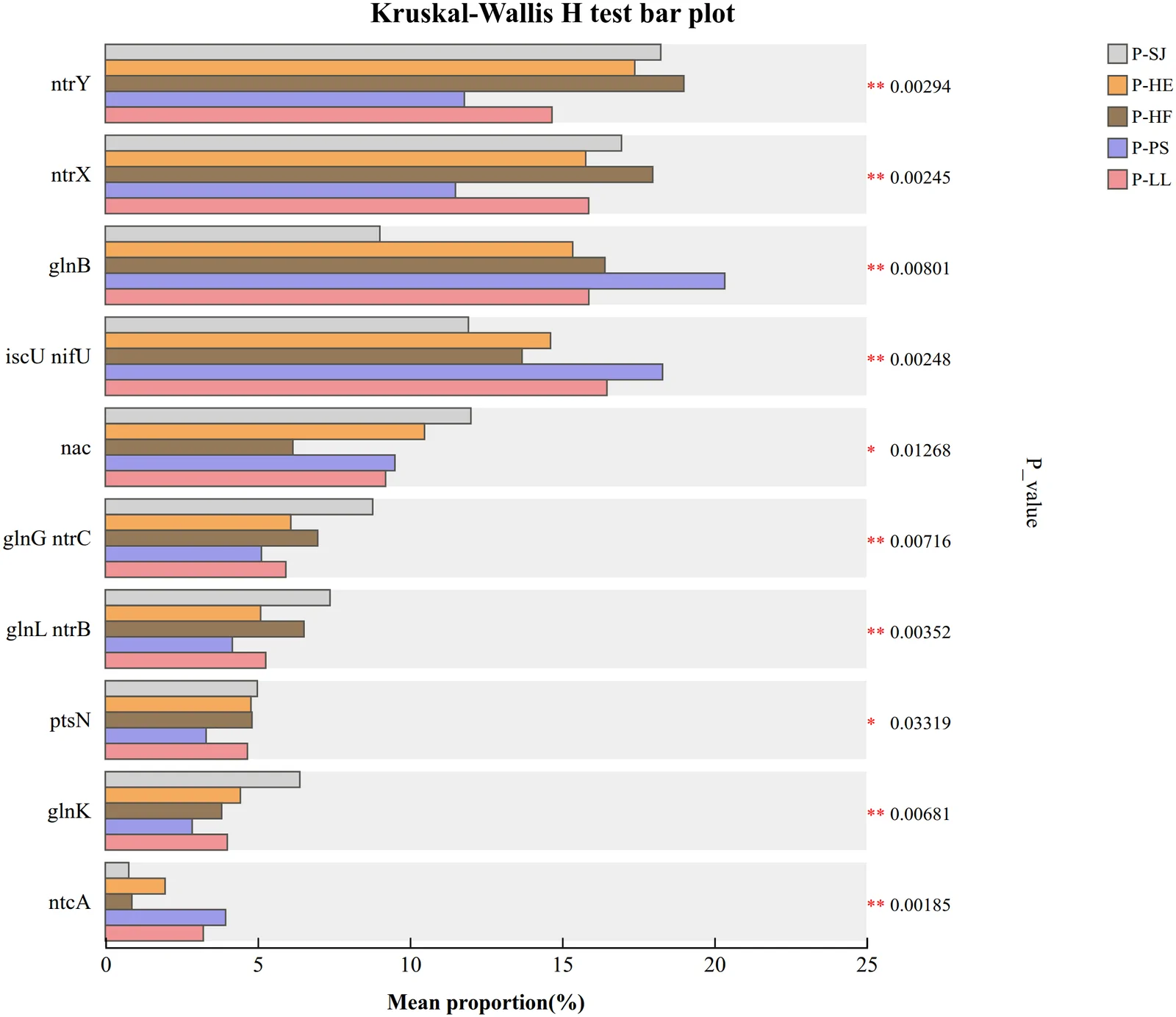
Differential abundance analysis of soil nitrogen-cycling functional genes under different herbaceous perennials.

**Figure 8** illustrates the redundancy analysis (RDA) biplot of soil chemical properties and nitrogen-cycling microbial functional genes, which was utilized to reveal the extent to which soil chemical indicators influence these genes. The results demonstrated that the ordination axes RDA1 and RDA2 explained 51.57% and 5.73% of the total variance, respectively, achieving a cumulative explanatory rate of 57.30%. This indicates that the environmental factors retained after collinearity screening effectively explained the distributional variances of the functional gene communities. Regarding the biplot clustering, the different herbaceous perennial groups were distinctly separated, highlighting significant differences in gene composition among the treatments. Based on the correlations between environmental factors (red lines) and functional genes (green lines), SMBN exerted the strongest influence on specific functional genes, such as *ntrY* (nitrogen regulation sensor histidine kinase) and *ntrX* (nitrogen regulation response regulator protein). It showed a significant positive correlation and primarily drove the community distribution of the P-SJ samples. Furthermore, the directional effects of environmental factors such as pH, TN, and NH_4_^+^-N were highly consistent. They exhibited strong correlations with genes like *ntcA* (global nitrogen regulator protein) and *glnB* (nitrogen regulatory protein P-II 1), exerting a robust regulatory effect on the community distributions of the P-PS and P-LL groups. Further analysis of the vector angles revealed that the extremely small angles between the rays of the functional genes (*ntrY* and *ntrX*) and SMBN indicated a significant positive correlation, corresponding to high abundances in the P-SJ group. Conversely, the acute angles between genes such as *ntcA* and *glnB* and environmental factors (pH, TN, and NH_4_^+^-N) suggested strong positive correlations, with intense responses primarily observed in the P-PS group. These results suggest that soil physicochemical properties, primarily represented by pH, TN, NH_4_^+^-N, and SMBN, are the core factors driving the alterations in the spatial distribution patterns of functional genes within this system.

**Figure 8:**
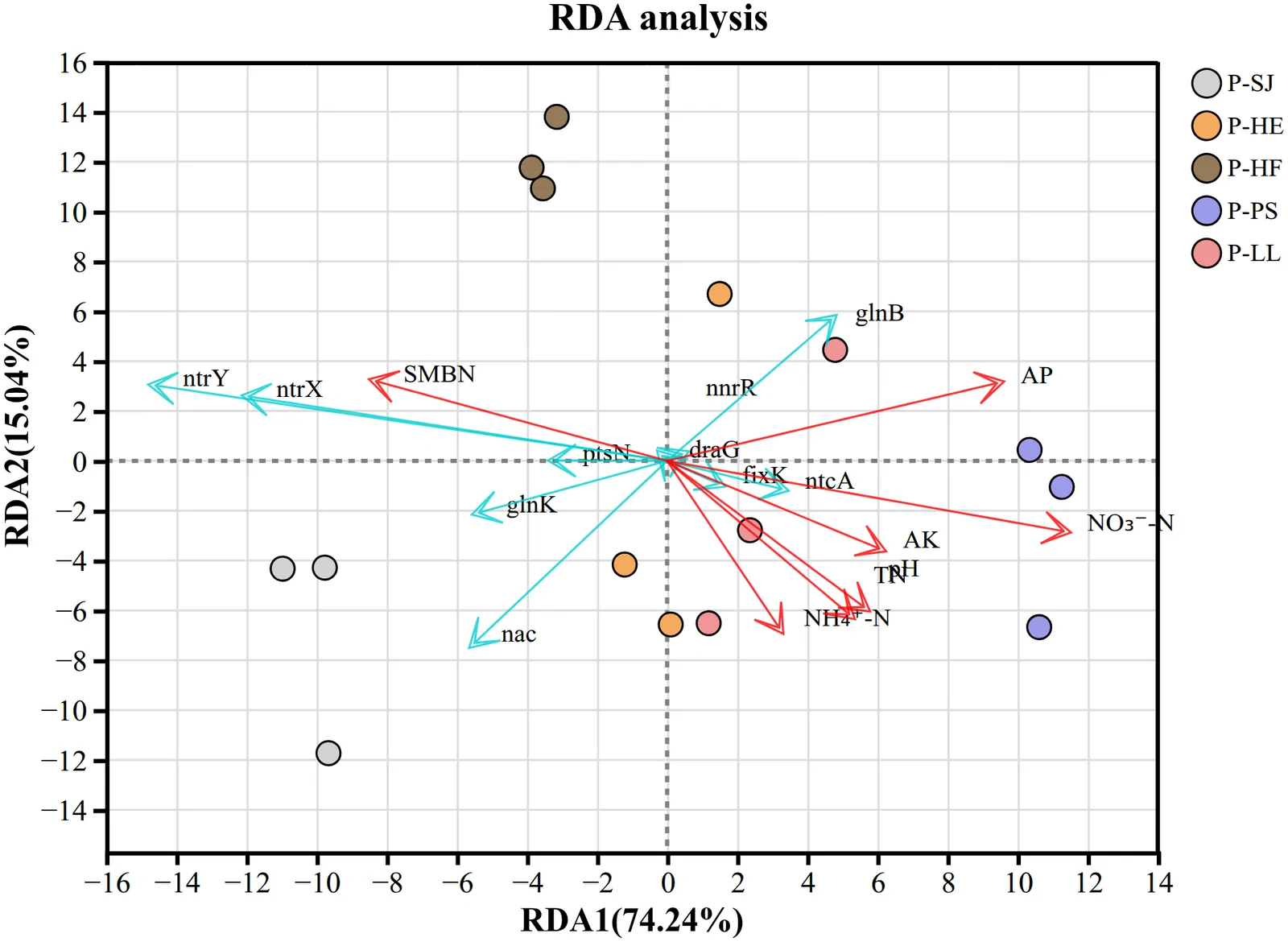
Redundancy analysis (RDA) of soil nitrogen-cycling functional genes under different herbaceous perennials.

As illustrated in **Figure 9**, the Spearman correlation analysis elucidated the differential impacts of soil physicochemical factors on the principal KEGG metabolic pathways. The results indicated that the two-component system experienced the most pronounced inhibitory effects from environmental factors, exhibiting a highly significant negative correlation with nitrate nitrogen (NO_3_^-^-N; *P* < 0.001) and significant negative correlations with total nitrogen (TN) and available phosphorus (AP) (*P* < 0.05). Additionally, the phosphotransferase system (PTS) also demonstrated a significant negative correlation with NO_3_^-^-N (*P* < 0.05). In terms of overall distribution trends, the pathway responses exhibited a distinct polarization. Core pathways—such as nitrogen metabolism, the nitrogen cycle, microbial metabolism in diverse environments, and global metabolism—generally displayed positive correlations with most physicochemical factors (e.g., NH_4_^+^-N, pH, and AK), but showed a consistent negative correlation with SMBN. In contrast, the mTOR signaling pathway, two-component system, and PTS pathways were positively correlated with SMBN, but negatively correlated with the majority of other nutrient factors. These findings suggest that elevated contents of available soil nutrients (particularly NO_3_^-^-N) may restrict specific microbial signal transduction and transmembrane transport processes, whereas these functional systems are closely associated with the microbial immobilization of their own biomass nitrogen.

**Figure 9:**
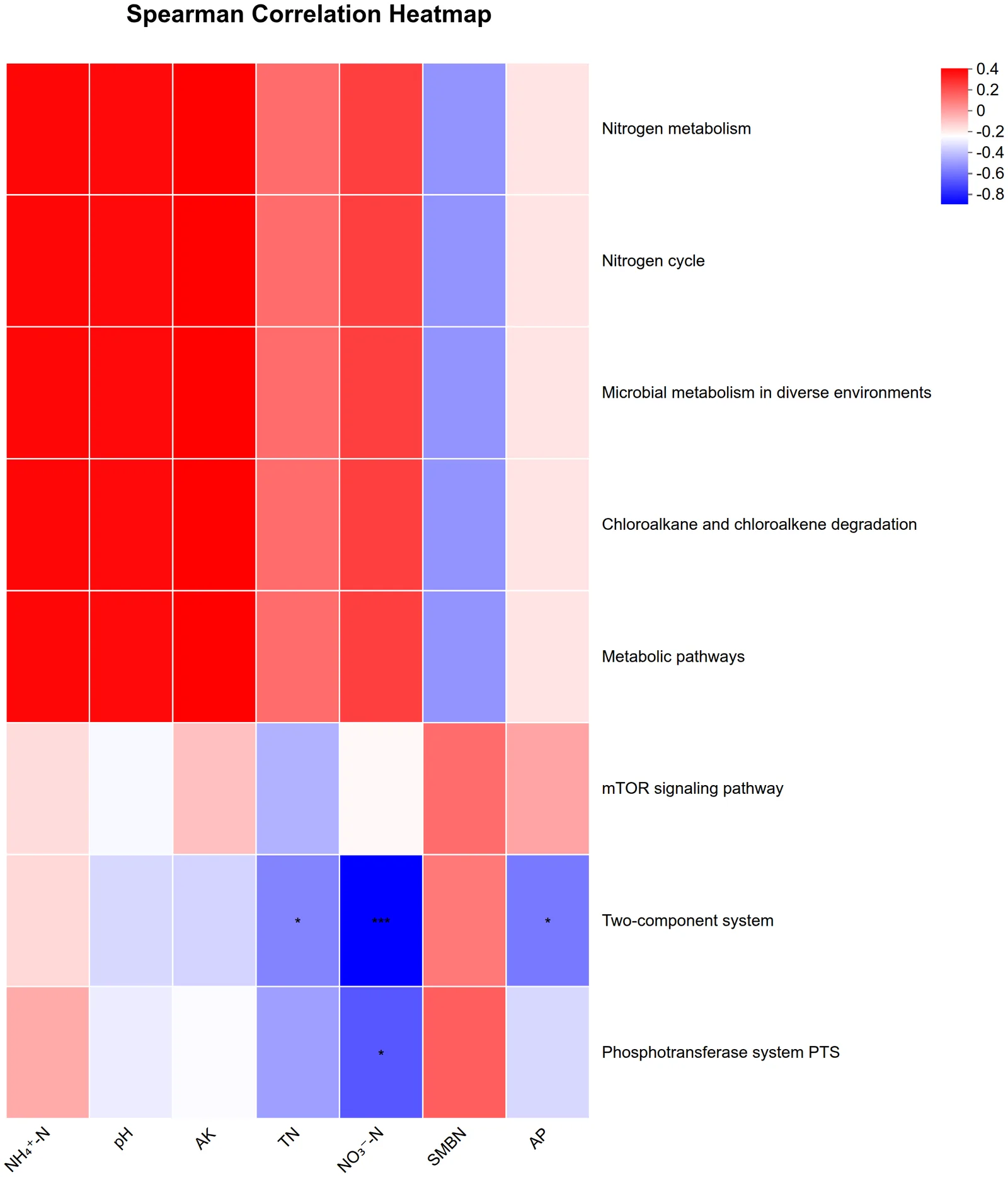
Spearman correlation heatmap between soil physicochemical factors and major KEGG functional pathways.

Building upon the RDA and Spearman correlation analyses, a Random Forest model was further employed to quantify the importance of individual soil physicochemical factors in driving the abundances of key nitrogen-cycling functional genes (**Figure 10**). The results revealed distinct variations in the predictive weights of different environmental factors for the key genes across various metabolic pathways. Regarding the core genes governing the ammonia assimilation pathway, total nitrogen (TN) and nitrate nitrogen (NO3^-^-N) emerged as the most critical driving factors for predicting the abundances of *GLUD1_2*/*gdhA* and *gltB*, respectively. Conversely, available potassium (AK) exhibited the highest Increase in Mean Squared Error (%IncMSE) when predicting the abundances of *gltD* and *glnA*. Within the predictive models for assimilatory nitrate reduction-related genes (e.g., *NRT2* and *narK*) and certain specific ammonia assimilation genes, factors such as pH, TN, and NO3^-^-N similarly demonstrated substantial contributions to the predictive importance. Overall, the contents of TN, AK, and NO3^-^-N acted as the primary limiting and regulatory factors driving the core microecological processes of nitrogen cycling within the current study system.

**Figure 10:**
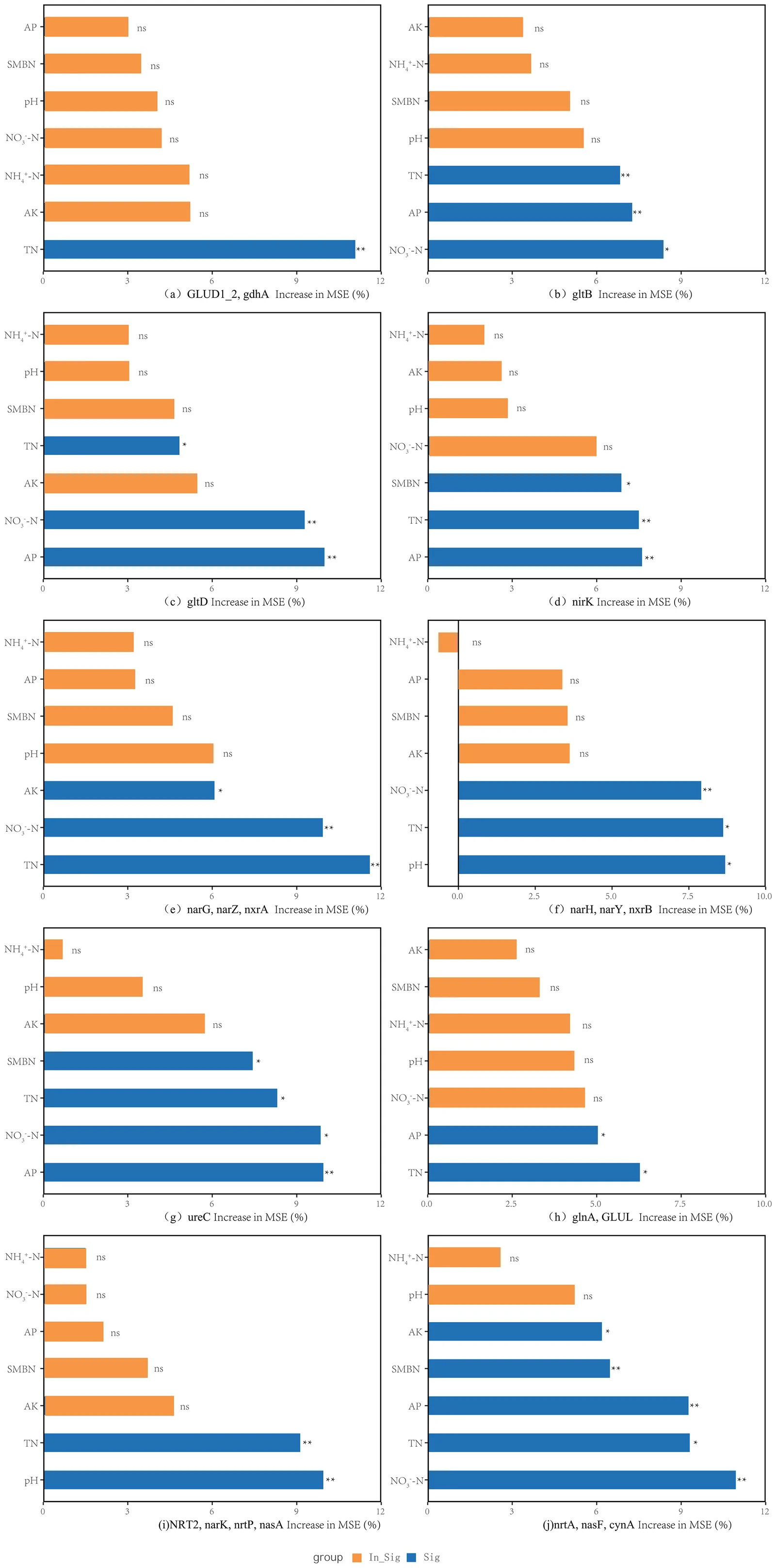
Random Forest importance ranking of soil physicochemical factors influencing the abundance of key nitrogen-cycling functional genes.

## 4 Discussion

### 4.1 Specific effects of different herbaceous perennials on soil physicochemical characteristics and nutrient availability

Regarding nutrient activation, the contents of available potassium (AK), total nitrogen (TN), and available phosphorus (AP) varied among the different floral treatments, with the P-LL treatment exhibiting a relatively higher comprehensive nutrient level. Previous studies have demonstrated that extensive plant root networks or the input of acidic substances can facilitate the turnover of insoluble minerals (Li et al., 2022). The high nutrient accumulation observed in the P-LL treatment may be attributed to such rhizosphere activities, which warrants future quantitative verification through the measurement of root exudates and extracellular enzyme activities. Concurrently, the P-HF treatment recorded the highest soil moisture content, demonstrating a robust water retention capacity (Batista et al., 2024). Compared to the one-sidedness of individual nutrient indicators, the Soil Multifunctionality Index (SMI) can comprehensively reflect the synergistic evolution of multiple ecosystem processes (Zheng et al., 2022; Fan et al., 2023). The SMI peaked in the P-LL treatment, indicating that it not only promotes the accumulation of individual nutrient components but also achieves the synergistic cycling and immobilization of diverse biogenic elements (e.g., C, N, P, and K) via complex rhizosphere microecological effects, thereby conferring superior comprehensive fertility characteristics to the basal soil. As this study did not include an unplanted blank control, it is difficult to completely exclude the influence of natural factors, such as climate fluctuations, on the soil background values. However, compared with the initial baseline data from May 2023, the TN and AK contents in the P-LL treatment increased from 0.80 g/kg and 113.1 mg/kg to 1.22 g/kg and 194.43 mg/kg, respectively, presenting a significantly distinct nutrient variation trend compared to other treatments such as P-HF. Under identical field management conditions, the pronounced divergence in nutrient variations among the treatments indicates that the observed nutrient immobilization and turnover were primarily driven by the species-specific rhizosphere effects of the different herbaceous perennials, rather than solely by soil spatial heterogeneity. Future studies should incorporate strict controls to accurately quantify the net effects of the plants.

In terms of nitrogen turnover, the P-PS treatment exhibited a prominent “high available nitrogen–low microbial biomass nitrogen” characteristic: its rhizosphere alkali-hydrolyzable nitrogen (AN) and ammonium nitrogen (NH_4_^+^-N) reached their maximum values of 120.96 mg/kg and 10.22 mg/kg, respectively, whereas the soil microbial biomass nitrogen (SMBN) dropped to the lowest level (32.06 mg/kg). When available nitrogen is limited, plants can stimulate microorganisms via carbon inputs to accelerate the mineralization of native soil organic matter (SOM), generating a positive priming effect (Koprivova and Kopriva, 2022). The nutrient distribution in the P-PS treatment aligns with the characteristics of this turnover paradigm; however, its actual underlying mechanism requires further verification using techniques such as isotope tracing. The generally low SMBN observed in most treatments (e.g., P-PS) is primarily constrained by the principles of plant-microbe resource competition (Xu et al., 2011). The high demand for available nitrogen during the vigorous plant growth stage can compress the assimilation space for microorganisms (Hao et al., 2025), and certain small-molecule metabolites may also inhibit microbial proliferation (Cheng et al., 2022). The phenomenon of high available nitrogen coupled with low SMBN in the P-PS treatment is consistent with the aforementioned plant-microbe competition mechanism, which can be corroborated in the future by quantitatively resolving plant nitrogen uptake and microbial nitrogen use efficiency via ^15^N isotope tracing. Furthermore, the relatively higher nitrate nitrogen content in the P-HE treatment reflects that different plants can reshape specific nitrogen-transformation microecological networks through differential root inputs (Liu et al., 2024).

### 4.2 Driving mechanisms of different herbaceous perennials on soil nitrogen-cycling microbial communities and functional genes

The root activities and litter inputs of herbaceous perennials significantly drove the changes in the diversity and spatial structure of the soil nitrogen-cycling microbial communities. This study found that different herbaceous perennials significantly altered the alpha and beta diversity of soil nitrogen-cycling microorganisms, reflecting the strong directional selection effect of the host on the rhizosphere microenvironment (Kuzyakov and Razavi, 2019). The bacterial community diversity was significantly highest in the P-SJ treatment, whereas the fungal community diversity peaked in the P-PS treatment. Principal coordinate analysis (PCoA) further confirmed that the treatments formed significant spatial niche separation. This is primarily because the species-specific root exudates of the different flowers reshaped specialized rhizosphere habitats as substrates, thereby filtering out specific adaptive microbial taxa (Fierer, 2017; Delgado-Baquerizo et al., 2024).

Regarding community composition, the core nitrogen-cycling taxa consisted of Pseudomonadota, Actinomycetota, and Acidobacteriota. Integrating the r/K ecological strategy theory, the succession of dominant communities profoundly reveals the plant-microbe nutrient coupling mechanisms. The relatively high abundance of r-strategy bacteria, such as Pseudomonadota, in the P-SJ and P-HE treatments indicates that their roots can release abundant soluble labile carbon sources, which favors the proliferation of fast-growing microorganisms (Veen et al., 2023; Guo et al., 2025). In contrast, the P-PS treatment exhibited a significantly higher relative abundance of K-strategy microorganisms, represented by Actinomycetota. These microorganisms possess the potential to degrade recalcitrant organic carbon, and their enhanced activity is often accompanied by the accelerated mineralization of organic nitrogen (Martinović et al., 2022). This is consistent with the high alkali-hydrolyzable nitrogen (AN) characteristics of the P-PS treatment, supporting the inference from a microbial taxonomic perspective that this treatment induces a rhizosphere priming effect. The higher abundance of K-strategy microorganisms and the accumulation of inorganic nitrogen logically align with the elevated AN characteristic observed in the P-PS soil; concurrently, the intensified plant-microbe resource competition can also explain its low SMBN phenomenon (Martinović et al., 2022). The related mechanisms still require verification through quantitative data, such as organic carbon degradation rates.

Beyond community composition, herbaceous perennials significantly reshaped the microbial co-occurrence network patterns. Topological properties indicated that the rhizosphere network under the P-PS treatment exhibited a relatively higher average degree and robustness, which aligned with its substantial enrichment of Actinobacteria (K-strategists) capable of degrading complex carbon sources. Ecological theory posits that K-strategist-dominated assemblages tend to form more tightly knit co-occurrence networks during resource competition, thereby enhancing the community’s resistance to environmental perturbations (Yuan et al., 2021). Therefore, we postulate that specific herbaceous perennials (e.g., P-PS) strengthen the synergistic interactions among core nitrogen-cycling taxa via distinct rhizosphere inputs. This highly connected network structure constitutes the crucial microecological foundation for sustaining its high-intensity organic nitrogen mineralization.

Alterations in the taxonomic structure of the community are inevitably accompanied by the reshaping of ecological functional potential. *ntrY* is a key gene for microorganisms to sense environmental nitrogen signals and initiate physiological responses. The relative abundance of the *ntrY* gene was highest and intra-group stable in the P-HF treatment, suggesting high nitrogen competition pressure or availability fluctuations in its rhizosphere, which prompted microorganisms to upregulate their sensing thresholds to enhance the capture of limited nitrogen sources. In the P-PS samples, the abundance of this gene was highly significantly lower and exhibited strong heterogeneity, indicating that under an abundant background of massive organic nitrogen mineralization, the reliance of the microbial community on environmental nitrogen sensing pathways had substantially weakened (Xia et al., 2024).

Collectively, these findings demonstrate that herbaceous perennials not only reshape the diversity and composition of soil nitrogen-cycling microorganisms via their root systems but also intervene in their environmental nitrogen sensing strategies at the functional gene level, thereby precisely driving the turnover processes of soil nitrogen pools at the microscale.

### 4.3 Regulatory characteristics of soil nitrification and denitrification processes driven by different herbaceous perennials

Soil nitrogen transformation heavily relies on the metabolic drive of microbial communities, whereas plant root exudates and microenvironmental characteristics serve as the core factors shaping the rhizosphere microbial functional network (Garcia et al., 2026; Zhang et al., 2023). This study demonstrated that the compositional patterns of soil nitrogen-cycling functional genes (KOs) were highly similar across the different herbaceous perennial treatments (**Figure 6**). None of the treatments altered the metabolic paradigm dominated by ammonia assimilation, indicating that the overall nitrogen metabolic network of the microbial community within this habitat possesses strong conservatism and functional redundancy (Nuccio et al., 2023; Sieradzki et al., 2023).

Within the ammonia assimilation pathway, the core genes responsible for assimilating free ammonia (*glnA* and *gltB*) exhibited exceptionally high abundances. In the rhizosphere microecology, intensive ammonia assimilation can generate intense competition for substrates (NH_4_^+^) with autotrophic nitrifying bacteria (Zha et al., 2024; Zhao et al., 2024). Although the dominant direction remained consistent, rhizosphere effects fine-tuned specific assimilation pathways: the P-HF treatment stimulated *glnA* abundance, the P-HE treatment displayed specifically higher relative *gltB* abundance, and the P-SJ treatment exhibited higher *gltD* levels. This differentiation indicates that the flowers can directionally select microbial taxa with a robust biological immobilization potential (Li et al., 2026; Sun et al., 2026). This strong immobilization effect limits the input of nitrification substrates at the source, effectively suppressing the potential nitrification rate. This represents a conservative strategy employed synergistically by plants and microbes to counteract nitrogen leaching (Chen et al., 2026; Wang et al., 2026).

The retention of nitrogen by the community is further reflected in the restriction of its gaseous loss; as the primary pathway for gaseous loss, the core denitrification gene (*nirK*) maintained consistently low levels (Wang S. et al., 2025), dropping to its lowest in treatments such as P-PS, which indicates that the rhizosphere did not induce hypoxic denitrification hotspots. This community functional feature perfectly aligns with the specific physical aeration status of the experimental soil: although the soil in this study area is classified as meadow black soil with a predominant clay loam texture—which theoretically facilitates the formation of localized hypoxic zones due to water retention—the soil moisture content (SMC) across all floral treatments during the measurement period remained generally very low (averaging only 9.88%, with a peak of 13.91%), leaving abundant pores filled with air. Such excellent aeration fundamentally deprives facultative anaerobic denitrifying bacteria of the hypoxic microniche required for their proliferation. This natural physical constraint on hypoxic habitats, coupled synergistically with the highly efficient ammonia assimilation maintained by the herbaceous perennials, further blocks the gaseous loss of nitrogen at the source. In summary, the physical hypoxic limitation works in synergy with the highly efficient ammonia assimilation of the herbaceous perennials (centered around *glnA* and *gltB*) to suppress the denitrification process at the source, thereby establishing a conservative “high immobilization–low loss” nitrogen metabolism paradigm.

### 4.4 Correlations and driving mechanisms among soil environmental factors, microorganisms, and functional genes

The redundancy analysis (RDA) results indicated that, following collinearity screening, physicochemical factors represented by pH, TN, NH_4_^+^-N, and SMBN were the core variables driving the spatial distribution patterns of nitrogen-cycling genes within this system (with the first two axes cumulatively explaining 57.30% of the variance) (Liu and Zhao, 2024). Among these, SMBN exhibited a significant positive correlation with the nitrogen regulation two-component system genes (*ntrY* and *ntrX*). The *ntrY*/*ntrX* system represents a key sensory pathway for microorganisms to perceive environmental nitrogen limitation and activate nitrogen uptake (Lira-Zumbado and Cotter, 2025). This tight coupling relationship suggests that higher levels of microbial nitrogen immobilization (SMBN) inherently intensified the competitive consumption of alkali-hydrolyzable nitrogen within the microenvironment, thereby forcing microorganisms to upregulate the abundance of environmental sensing and regulatory genes to sustain survival. Conversely, NO_3_^-^-N demonstrated a strong positive correlation with global nitrogen regulatory genes (*ntcA* and *glnB*) (Selim et al., 2024). This indicates that herbaceous perennials can regulate the localized supply of nitrate nitrogen via rhizosphere effects, which subsequently directly modulates the primary nitrogen metabolism and assimilation rates of specific dominant microbial taxa (Ma et al., 2025). Integrating the variable importance ranking from the Random Forest model, this study further elucidated how plant species specifically regulate nitrogen-cycling metabolic pathways by altering particular soil chemical properties. The model corroborated that total nitrogen (TN), nitrate nitrogen (NO3^-^-N), and available potassium (AK) serve as the paramount direct drivers determining the abundances of core ammonia assimilation genes (e.g., *glnA* and *gltB*) and nitrate reduction-related genes. This indicates that different herbaceous perennials do not randomly alter the overall soil microenvironment; instead, they directionally reshape specific available nutrient pools (particularly TN and NO3^-^-N) in the rhizosphere via species-specific root activities. Such plant-mediated divergence in specific physicochemical traits exerts a profound environmental filtering effect, thereby directionally screening and regulating microbial taxa that harbor specific functional genes. For instance, the distinct nutrient enrichment profiles exhibited by P-LL and P-PS constitute the critical physicochemical foundation enabling their directional intervention in the ammonia assimilation intensity and nitrogen-sensing thresholds within the underlying soil.

Further pathway analysis (**Figure 9**) revealed a “polarized” driving mechanism of environmental factors on microbial functions. Global nitrogen-cycling and metabolic pathways were generally positively correlated with available nutrients (e.g., NH_4_^+^-N and AK), whereas the two-component system and the PTS transmembrane transport pathways were highly significantly inhibited by NO_3_^-^-N (*P* < 0.001). Such response divergence at the functional network level profoundly reflects the ecological strategy trade-offs of soil microorganisms in coping with environmental fluctuations (Jaeger et al., 2024). When the environment is rich in available nutrients such as NO_3_^-^-N, microorganisms tend to adopt a “copiotrophic (r-strategy)” mode, prioritizing energy allocation toward overall nitrogen metabolism and rapid proliferation (Yin et al., 2025). Conversely, when alkali-hydrolyzable nitrogen is relatively deficient and environmental nitrogen is primarily immobilized by the microbial community (SMBN), microorganisms shift toward an “oligotrophic (K-strategy)” mode (Geng et al., 2026). In this state, they must upregulate environmental signal transduction (the two-component system) and high-affinity substrate transport (PTS) to enhance their competitive ability for trace free nutrients (Ren, 2026).

## 5 Conclusions

This study elucidates the mechanisms by which different herbaceous perennials reshape soil microecology and nitrogen-cycling functions in urban habitats of Northeast China. The herbaceous perennials exhibited two distinctly divergent ecological adaptation strategies in the rhizosphere. The first is the “nutrient-retention” strategy, represented by P-LL, which yielded the highest contents of total nitrogen, available phosphorus, and available potassium, demonstrating a high-level soil multifunctionality index (SMI). The second is the “turnover-driven” strategy, represented by P-PS. Through a cascade pathway of “complex carbon inputs – high abundance of K-strategist Actinobacteria – stimulated organic matter mineralization,” P-PS triggered a potent rhizosphere nitrogen mineralization priming effect and constructed a microbial co-occurrence network with enhanced connectivity and robustness. This mechanism facilitated a massive release of available nitrogen, enabling the plant to achieve absolute dominance in microecological nitrogen competition (as indicated by the lower level of soil microbial biomass nitrogen).At the functional gene level, despite the r/K strategy divergence within the communities, the nitrogen-cycling genes across all treatments exhibited a high degree of ecological conservatism. All floral treatments were predominantly centered on ammonia assimilation genes (*glnA* and *gltB*) while maintaining an extremely low denitrification potential (*nirK*), successfully establishing a “high-retention, low-loss” nitrogen metabolic paradigm. Furthermore, comprehensive multivariate and Random Forest model analyses corroborated that physicochemical factors—typified by pH, TN, NH_4_^+^-N, and SMBN—drove the spatial distribution patterns of the functional genes, among which SMBN exerted the strongest positive regulation on the nitrogen-sensing regulator gene (*ntrY*). Meanwhile, TN, nitrate nitrogen (NO_3_^-^-N), and available potassium (AK) emerged as the most critical direct drivers determining the directional divergence of the core ammonia assimilation pathways.In conclusion, for the establishment of urban green spaces and the scientific management of soil nutrients in cold regions, we recommend the targeted configuration of superior floral species such as P-LL (for fertility retention) and P-PS (for nutrient turnover) based on their specific rhizosphere microecological characteristics. This approach will optimize underlying nutrient cycling efficiency and foster enhanced overall microecological functions.

## Data Availability

The datasets presented in this study can be found in online repositories. The names of the repository/repositories and accession number(s) can be found in the article/supplementary material.
